# Methylophiopogonanone A, an *Ophiopogon* homoisoflavonoid, alleviates high-fat diet-induced hyperlipidemia: assessment of its potential mechanism

**DOI:** 10.1590/1414-431X20199201

**Published:** 2020-03-02

**Authors:** Zhao Li, Ying-Ying Wu, Bei-Xin Yu

**Affiliations:** Center for Translational Medicine of the First Affiliated Hospital, Sun Yatsen University, Guangzhou, China

**Keywords:** Ophiopogon japonicus, PPARα, High-fat diet, Lipid lowering, Dyslipidemia

## Abstract

Methylophiopogonanone A (MO-A), a homoisoflavonoid extracted from *Ophiopogon japonicus*, has been shown to attenuate myocardial apoptosis and improve cerebral ischemia/reperfusion injury. However, the hypolipidemic effects remain unknown. This study was performed to investigate a potential hypolipidemic effect of MO-A in hyperlipidemia rats, as well as its underlying mechanism of action. A rat model of hyperlipidemia was induced by a high-fat diet (HFD). Animals were randomly divided into three groups (n=8/group): normal control group (NC), HFD group, and HFD+MO-A (10 mg·kg^-1^·d^-1^) treatment group. The effects of MO-A on serum lipids, body weight, activity of lipoprotein metabolism enzyme, and gene expression of lipid metabolism were evaluated in HFD-induced rats. In HFD-induced rats, pretreatment with MO-A decreased the body weight gain and reduced serum and hepatic lipid levels. In addition, pretreatment with MO-A improved the activities of lipoprotein lipase and hepatic lipase in serum and liver, down-regulated mRNA expression of acetyl CoA carboxylase and sterol regulatory element-binding protein 1c, and up-regulated mRNA expression of low-density lipoprotein receptor and peroxisome proliferator-activated receptor α in the liver. Our results indicated that MO-A showed strong ability to ameliorate the hyperlipidemia in HFD-induced rats. MO-A might be a potential candidate for prevention of overweight and dyslipidemia induced by HFD.

## Introduction

Hyperlipidemia is a lipid metabolic disorder disease, which is considered the major pathogenic factor for progression and development of cardiovascular diseases, such as myocardial infarction, cerebrovascular diseases, stroke, diabetes mellitus, and atherosclerosis (1). It is a group of disorders characterized by increased triglycerides, high cholesterol and plasma lipid levels, and usually coexists with obesity (2). Currently, the treatment of lipid metabolic disorder disease represents a huge cost in both developing and developed countries, and it has become a serious social problem (3). A decrease in abnormal lipid level is an effective approach for prevention or treatment of lipid metabolic disorder disease. Although some chemical drugs are presently used to treat hyperlipidemia, the clinical use of chemical drugs is limited because of their potential adverse effects, such as kidney function damage, myopathy, liver injury, etc (4,5). In recent years, an increasing interest in the investigation of pharmacologic effects of Chinese herbal medicine has been occurring. Bioactive ingredients derived from herbal medicines are considered efficient therapies to alleviate lipid metabolic disorder diseases because they have a high potency against dyslipidemia with few adverse effects (6).

*Ophiopogonis Radix*, the dried tuber root of *Ophiopogon japonicus* (Thunb.) Ker-Gawl (Liliaceae), has been widely used to treat cardiovascular, diabetes, and chronic inflammatory diseases for many years (7,8). Homoisoflavonoids, polysaccharides, and steroidal sapogenins are the main bioactive constituents of *Ophiopogon japonicus*, and methylophiopogonanone A (MO-A) is the major homoisoflavonoid present in *Ophiopogon japonicus* (9). Accumulating evidence has revealed that MO-A attenuates myocardial apoptosis and improves cerebral ischemia/reperfusion injury (10,11). However, to the best of our knowledge, it remains unknown whether MO-A possesses lipid lowering effects in rats fed a high-fat diet (HFD).

Thus, in our study, we aimed to investigate the potential effect of MO-A supplement on alleviating hyperlipidemia and improving lipid metabolic disorders in an HFD-induced obese rat model. The lipid metabolism-related genes were also evaluated to explore the underlying mechanism of MO-A's activities on lipid metabolism.

## Material and Methods

### Material and chemicals

MO-A (purity >98%) was purchased from Ronghe Technology Co., LTD (China). All other chemical reagents were of analytical grade purchased from Aladdin Reagent Co. (China).

### Animals and experimental design

Six-week-old Sprague-Dawley male rats (200±20 g) were purchased from the Laboratory Animal Center of Guangdong (China) and kept in the Experimental Animal Center of Sun Yatsen University under standard laboratory environments (humidity 50–60%, air temperature 20–25°C, and 12-h light/dark cycle), with free access to food and water. The rat experiments were approved by the animal Ethics Committee and carried out in accordance with local institutional regulations.

All rats were fed with standard chow for seven days to adapt to the laboratory environment and then randomly separated into three groups according to body weight: NC (standard chow, n=8), HFD (high-fat diet, n=8), and HFD+MO-A (high-fat diet, n=8). NC were fed basic diets and the remaining two groups were fed HFD as shown in Table 1 for eight weeks of the experimental period. NC and HFD groups were administered with the same amount of water intragastrically, while HFD+MO-A group was administered 10 mg·kg^-1^·d^-1^ of MO-A intragastrically for 8 consecutive weeks. The dose of MO-A (10 mg·kg^-1^·d^-1^) was determined according to the previous study and our preliminary experiments (10). Body weight and food intake were recorded during the entire experiment. The food efficiency ratio was calculated by the following formula: weight gain / food intake × 100%.

**Table 1.** Composition of the diets (%).

Ingredient	High-fat diet	Basic diet
Soybean meal	12	20
Corn meal	25	30
Wheat flour	15	19
Wheat bran	23	26
Fish meal	4	5
Cholesterol	3	0
Methylthiouracil	0.2	0
Sodium cholate	0.3	0
Lard	7.5	0
Yolk powder	10	0

### Assay of fecal lipids

At the end of the experiment, excrements of rats were collected, lyophilized, and weighed. Total lipids in excrements were extracted according to a previous report (12). Briefly, the feces were powdered and 0.2 g of feces was extracted with 10 mL methanol/chloroform (1:2, v/v) for 30 min by ultrasonic treatment. The supernatant was collected and total lipids were obtained by vacuum drying.

### Sample collection

After the last experiment, the rats were fasted over-night and blood samples were collected from the retro-orbital sinus using a capillary tube. Then, blood was centrifuged at 8000 *g* for 15 min at 4°C to obtain serum sample and stored at −20°C until analysis. All rats were executed by cervical dislocation. The liver tissues and visceral and abdominal adipose tissues were quickly harvested and washed using cold physiological saline and wiped with filter paper. After weighing, the liver tissues were homogenized (10%, w/v) in cold physiological saline and then centrifuged at 5000 *g* for 15 min at 4°C. The supernatant was collected and stored at −20°C for further analysis. The liver index was calculated by the following formula: liver weight / body weight × 100%.

### Lipid profile analysis

The levels of triacylglycerol (TG), total cholesterol (TC), high-density lipoprotein cholesterol (HDL-C), low-density lipoprotein cholesterol (LDL-C), hepatic TC, hepatic TG, aspartate transaminase (AST), and alanine aminotransferase (ALT) were assayed with a commercial assay kit (Nanjing Jiancheng Bioengineering Institute, China), according to the manufacturer's instructions. Measurements of lipoprotein lipase (LPL) and hepatic lipase (HL) activities were performed in serum and liver tissue.

The total protein of liver homogenate was assayed using the Coomassie brilliant blue method (13). The activities of LPL and HL were assayed using a commercial kit (Nanjing Jiancheng Bioengineering Institute), according to the manufacturer's instructions. Briefly, 100 µL of sample solution was incubated with a solid antibody at 37°C for 60 min in the semi-micro cuvettes. After washing with phosphate buffer, the antibody labeled with horseradish peroxidase was mixed and cultured at 4°C for 30 min. Then, chromogen was added and cultured at room temperature for 30 min. After 1 N H_2_SO_4_ was added, absorbance was measured using a microplate reader (Thermo Fisher Scientific, USA) at 450 nm.

### RNA isolation and quantitative real-time polymerase chain reaction (qRT-PCR)

Total RNA was extracted from frozen rat liver tissues using Trizol Reagent (Invitrogen, USA), following the manufacturer's protocol. cDNA synthesis was carried out using the First Strand cDNA Synthesis kit (Thermo Fisher Scientific). Real-time PCR was carried out using a SYBR Green qPCR Master Mix kit (Thermo Fisher Scientific). The qPCR was performed in duplicate, the parameter of RT-PCR amplification reaction was as follows: 40 cycles of 95°C for 10 s, 60°C for 15 s, and 72°C for 30 s, with the primer sequences shown in Table 2. Target mRNA expression of each sample was normalized to endogenous control gene GAPDH.

**Table 2.** Sequences of primers used in present study.

Gene	Forward primer	Reverse primer
ACC	5′-ACACTGGCTGGCTGGACAG-3′	5′-CACACAACTCCCAACATGGTG-3′
SREBP-1C	5′-CCCTGCGAAGTGCTCACAA-3′	5′-GCGTTTCTACCACTTCAGGTTTCA-3′
LDLR	5′-CCAACCTGAAGAATGTGGTG-3′	5′-CAGGTCCTCACTGATGATGG-3′
PPARα	5′-GGAAACTGCCGACCTCAAAT-3′	5′-AACGAAGGGCGGGTTATTG-3′
GAPDH	5′-GAACGGGAAGCTCACTGGC-3′	5′-GCATGTCAGATCCACAACGG-3′

### Western blotting

The liver tissue was homogenized in the lysate buffer. The lysate was centrifuged at 10,000 *g* for 15 min at 4°C. The content of total protein was determined using a protein quantitation kit (Nanjing Jiancheng Bioengineering Institute). Protein samples were separated by 12% SDS-polyacrylamide gels, and then transferred to a polyvinylidene fluoride membrane. After blocking in 5% defatted milk for 1 h at room temperature, the membrane was probed with the following primary antibodies overnight at 4°C (diluted 1:1,000): acetyl CoA carboxylase (ACC; Cell Signaling Technology, USA), low-density lipoprotein receptor (LDLR; Cell Signaling Technology), peroxisome proliferator-activated receptor-α (PPARα; Cell Signaling Technology), sterol regulatory element-binding protein 1c (SREBP-1c; Cell Signaling Technology), carnitine palmitoyltransferase-1 (CPT-1; Cell Signaling Technology), and GAPDH (Cell Signaling Technology). The membrane was washed with TBST and subsequently probed with horseradish peroxidase-linked secondary antibodies for 1 h at room temperature. Visualization was carried out using a chemiluminescence kit (Amersham, USA). Immunoreactive bands were quantified using ImageJ image analysis software (National Institutes of Health, USA).

### Measurement of CPT-1 and peroxisomal fatty acid oxidase (FAO) activities in liver tissue

The frozen liver tissue was minced and homogenized with cold physiological saline (1/5, w/v) and then centrifuged at 5,000 *g* for 15 min at 4°C. The activity of CPT-1 was measured in these supernatants spectrophotometrically (14). Briefly, 50 µL of cleared supernatant and 50 µL of Tris-HCl-DTNB buffer was transferred to semi-micro cuvettes. After incubation at 30°C for 5 min, 50 µL of palmitoyl-CoA was transferred to cuvettes. Then, 5 µL of L-carnitine was added, and immediately measured by photometric measurement. The FAO activity in these supernatants was determined using a commercial kit (Jiangxi Jianglan Pure Biological Reagent Co. LTD, China), according to the manufacturer's protocol. Briefly, 50 µL of the supernatant was mixed with the 100 µL of antibodies labeled with horseradish peroxidase at 37°C for 1 h. After the 50 µL of stop buffer was added, absorbance was assayed using a microplate reader at 450 nm. The content of protein in the supernatant was determined using a protein quantitation kit (Nanjing Jiancheng Bioengineering Institute).

### Statistical analysis

Experimental results are reported as means±SD and the experiments were replicated twice. Differences between groups were compared by GraphPad Prism software (GraphPad software, Inc., USA) using one-way ANOVA with Tukey's multiple comparison test. P<0.05 was considered as statistically significant.

## Results

### Effects of MO-A on food efficiency ratio, visceral fat, abdominal adipose tissue, and fecal lipids

**Figure 1.**
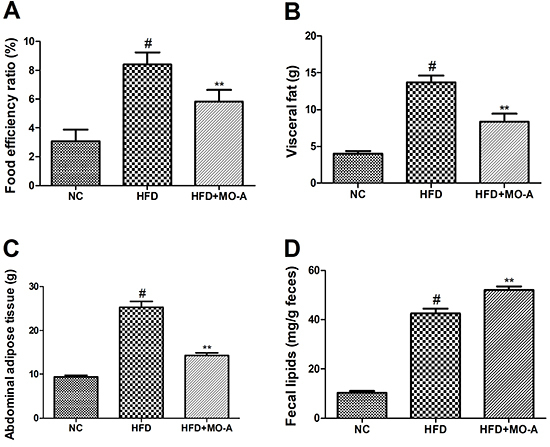
Effect of methylophiopogonanone A (MO-A) on **A**, food efficiency ratio **B**, visceral fat, **C**, abdominal adipose tissue, and **D**, fecal lipids in hyperlipidemic rats. Data are reported as means±SD, n=8 rats in each group. ^#^P<0.05, compared with normal control (NC) group; **P<0.01, compared with high-fat diet (HFD) group (ANOVA).

As shown in Figure 1, the food efficiency ratio, visceral fat, and abdominal adipose tissue in the HFD group were higher than those in the NC group (P<0.05). However, after administration of MO-A for eight weeks, the increase of the food efficiency ratio, visceral fat, and abdominal adipose tissue was markedly ameliorated compared with those in the HFD group (P<0.01). In addition, the content of fecal lipids in the HFD group was higher than that in the NC group (P<0.05). However, after administration of MO-A for eight weeks, the increase of fecal lipids was markedly accelerated compared to the HFD group (P<0.01).

### MPO-A inhibited weight gain in HFD-induced hyperlipidemia rats

**Figure 2.**
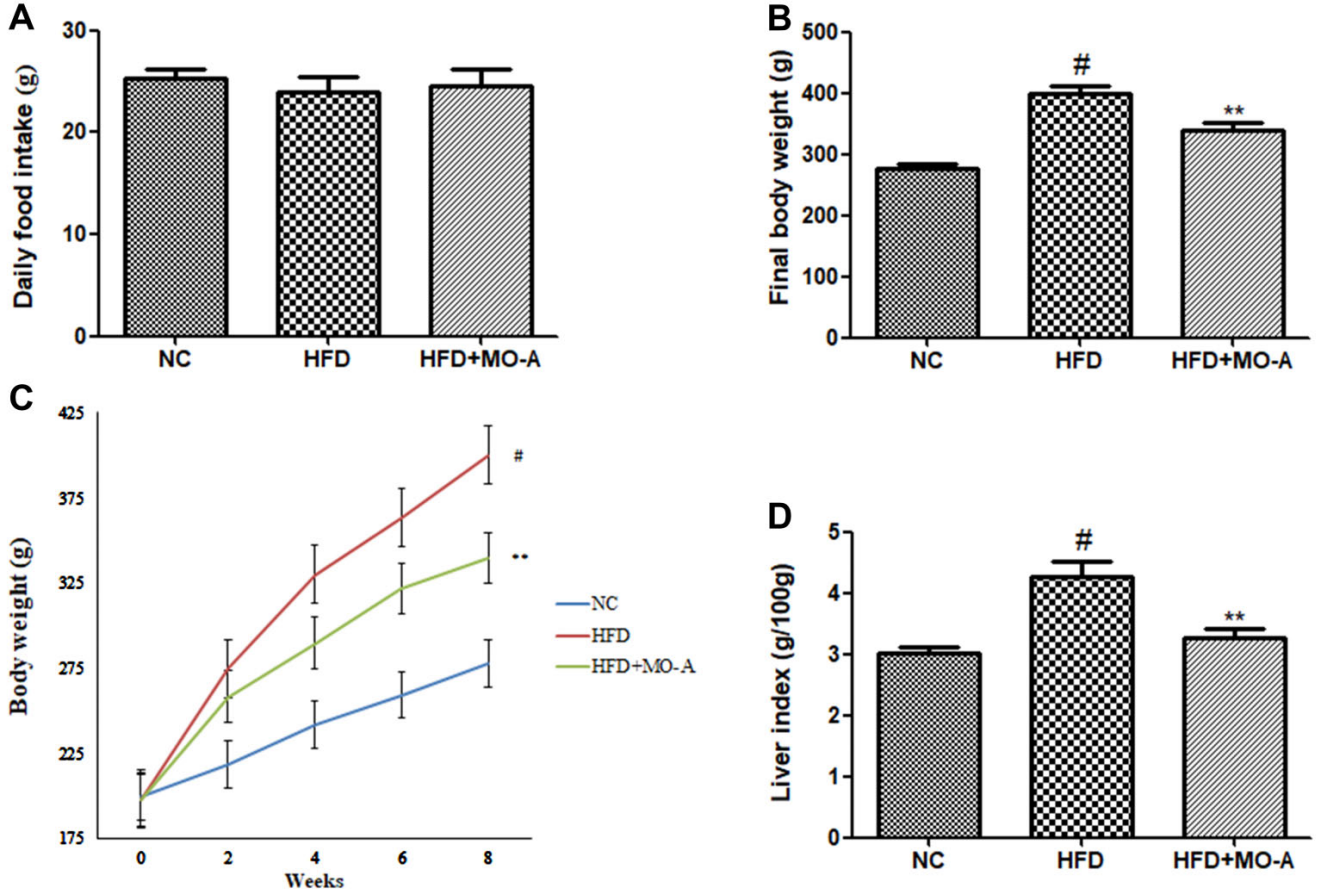
Effect of methylophiopogonanone A (MO-A) on **A**, daily food intake, **B**, final body weight, **C**, body weight, and **D**, liver index in hyperlipidemic rats. Data are reported as means±SD, n=8 rats in each group. ^#^P<0.05, compared with normal control (NC) group; **P<0.01, compared with high-fat diet (HFD) group (ANOVA).

As shown in Figure 2, after 8 weeks of treatment, the final body weight in the HFD group was higher than that in the NC group (P<0.05), while the final body weight in the MO-A group was significantly lower compared to the HFD group (P<0.05). The results indicated that supplementation with MO-A effectively prevented the body weight gain in HFD-induced hyperlipidemia rats. Since the suppression of body weight gain could be caused by a decrease of food intake, the food intake of the rat was recorded. The food intake of the MO-A group was not different from that of the HFD group, indicating that the decrease of body weight was due to the effects of MO-A and not from lower energy intake. As shown in Figure 2D, after eight weeks of treatment, the liver index in the HFD group was higher than that in the NC group (P<0.05), while the liver index in the MO-A group was lower compared to the HFD group (P<0.05).

### Effect of MO-A on the serum lipid profile in hyperlipidemia rats

**Figure 3.**
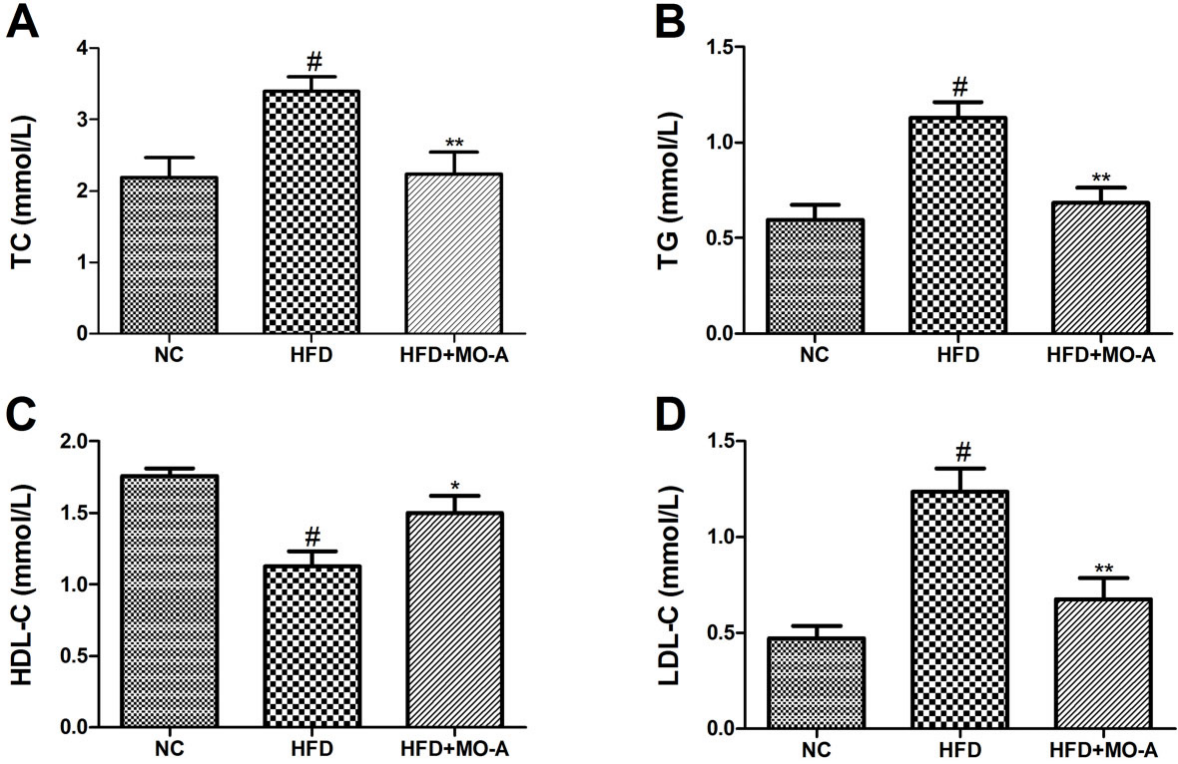
Effect of methylophiopogonanone A (MO-A) on serum levels of **A**, total cholesterol (TC), **B**, triacylglycerol (TG), **C**, high-density lipoprotein cholesterol (HDL-C), and **D**, low-density lipoprotein cholesterol (LDL-C) in hyperlipidemia rats. Data are reported as means±SD, n=8 rats in each group. ^#^P<0.05, compared with normal control (NC) group; *P<0.05, **P<0.01, compared with high-fat diet (HFD) group (ANOVA).

We measured the lipid profiles of serum samples from the different groups to investigate the effect of MO-A on dyslipidemia. As shown in Figure 3, administration of HFD to rats for eight weeks resulted in an increase in serum TC, TG, and LDL-C levels, and a decrease in serum HDL-C level compared to the NC group (P<0.01). The results indicated that a hyperlipidemic rat model was successfully induced. After administration of MO-A for eight weeks, the levels of TC, TG, and LDL-C in the MO-A group were lower compared to the HFD group (P<0.01). Furthermore, compared with the HFD group, the level of HDL-C was higher by oral administration MO-A for eight weeks (P<0.05), indicating that MO-A improved dyslipidemia in hyperlipidemia rats.

### Effect of MO-A on hepatic lipid levels in hyperlipidemia rats

**Figure 4.**
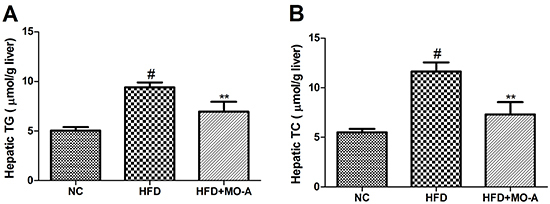
Effect of methylophiopogonanone A (MO-A) on hepatic lipid levels of **A**, triacylglycerol (TG) and **B**, total cholesterol (TC) in hyperlipidemia rats. Data are reported as means±SD, n=8 rats in each group. ^#^P<0.05, compared with NC group; **P<0.01, compared with high-fat diet (HFD) group (ANOVA).

Since the liver is an important site for lipid metabolism, we also assayed the hepatic TC and TG levels in hyperlipidemia rats. As shown in Figure 4, administration of HFD to rats for eight weeks resulted in an increase in hepatic TC and TG levels compared to the NC group (P<0.01). The results indicated that a hyperlipidemia rat model was successfully induced by HFD. After administration of MO-A for eight weeks, the levels of hepatic TG and TC were lower compared to the HFD group (P<0.01), indicating that MO-A reduced lipid accumulation in the liver tissue of hyperlipidemia rats.

### Effect of MO-A on serum and hepatic LPL and HL activities in hyperlipidemia rats

**Figure 5.**
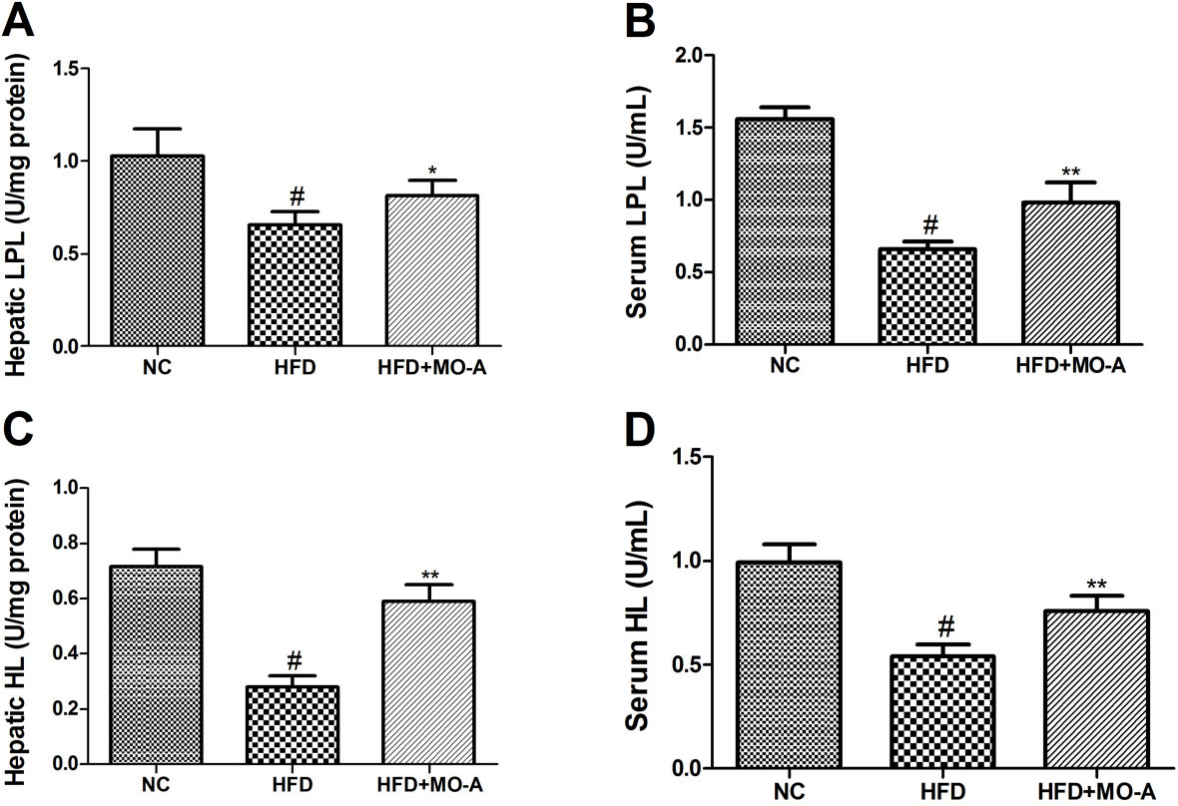
Effect of methylophiopogonanone A (MO-A) on hepatic **A**, lipoprotein lipase (LPL) and **C**, hepatic lipase (HL) activities and serum **B**, LPL and **D**, HL activities in hyperlipidemia rats. Data are reported as means±SD, n=8 rats in each group. ^#^P<0.05, compared with normal control (NC) group; *P<0.05, **P<0.01, compared with high-fat diet (HFD) group (ANOVA).

As shown in Figure 5, administration of HFD to rats for eight weeks resulted in a decrease in HL and LPL activities compared to the NC group (P<0.01). After administration of MO-A for eight weeks, the activities of HL and LPL in the MO-A group were higher compared to the HFD group (P<0.05 or P<0.01).

### Effect of MO-A on hepatic dysfunction in hyperlipidemia rats

**Figure 6.**
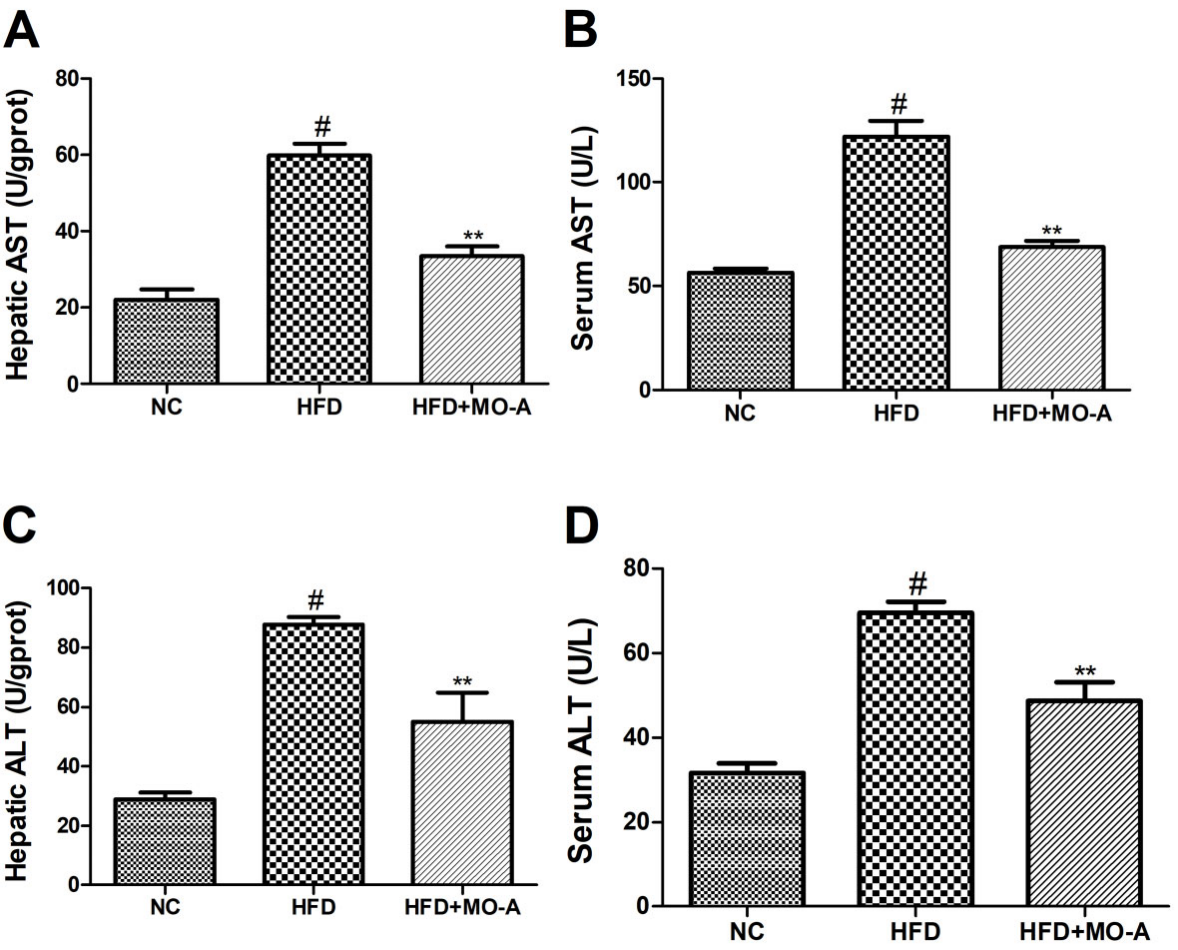
Effect of methylophiopogonanone A (MO-A) on **A** and **B**, aspartate transaminase (AST) and **C** and **D**, alanine aminotransferase (ALT) levels in hyperlipidemia rats. Data are reported as means±SD, n=8 rats in each group. ^#^P<0.05, compared with NC group; **P<0.01, compared with high-fat diet (HFD) group (ANOVA).

The serum and hepatic levels of AST and ALT were measured to evaluate the hepatotoxicity of MO-A in hyperlipidemia rats. As shown in Figure 6, both the serum and hepatic levels of ALT and AST in the HFD group were increased compared to the NC group (P<0.01). However, eight weeks of MO-A treatment decreased AST and ALT levels (P<0.01), indicating the beneficial effects of MO-A treatment on hepatic dysfunction in hyperlipidemia rats.

### Effect of MO-A on hepatic mRNA and protein expression of SREBP-1c, ACC, LDLR, and PPARα

**Figure 7.**
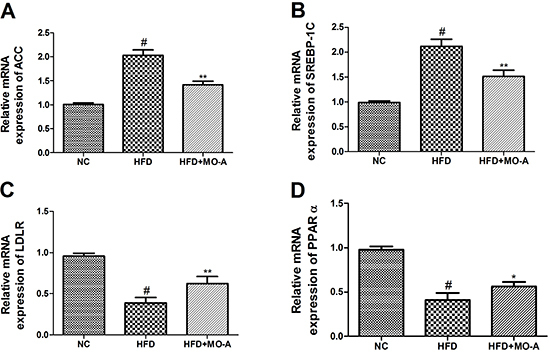
Effect of methylophiopogonanone A (MO-A) on hepatic mRNA expression of **A**, CoA carboxylase (ACC), **B**, sterol regulatory element-binding protein 1c (SREBP-1C), **C**, low-density lipoprotein receptor (LDLR), and **D**, peroxisome proliferator-activated receptor α (PPARα) in hyperlipidemia rats. Data are reported as means±SD, n=8 rats in each group. ^#^P<0.05, compared with normal control (NC) group; *P<0.05, **P<0.01, compared with high-fat diet (HFD) group (ANOVA).

**Figure 8.**
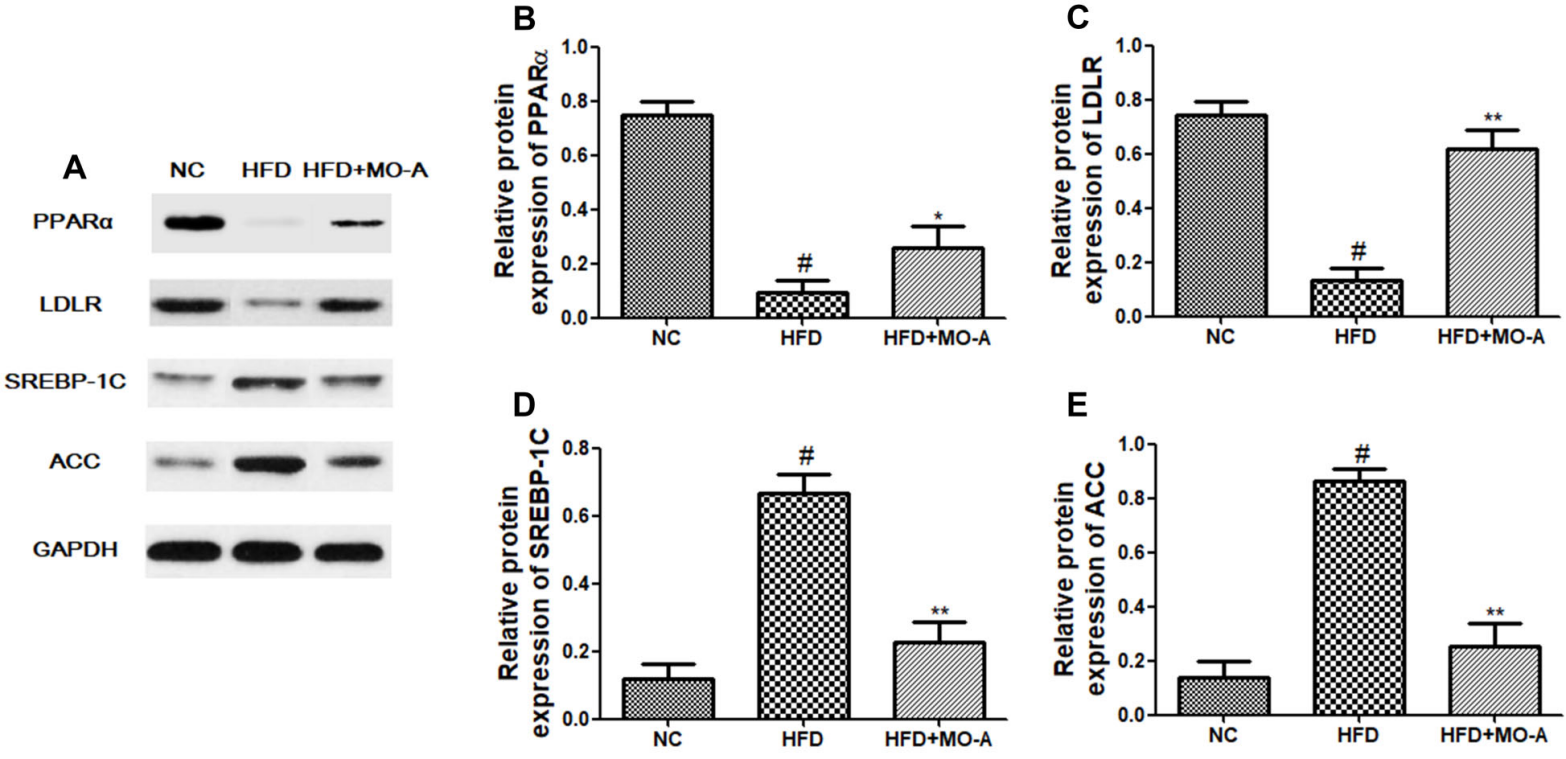
Expression of proteins in the livers of hyperlipidemia rats. **A**, Western blotting images of peroxisome proliferator-activated receptor α (PPARα), low-density lipoprotein receptor (LDLR), sterol regulatory element-binding protein 1c (SREBP-1C), and CoA carboxylase (ACC) protein expression. Relative expression of **B**, PPARα protein, **C**, LDLR protein, **D**, SREBP-1C protein, and **E**, ACC protein. Data are reported as means±SD, n=8 rats in each group. ^#^P<0.05, compared with normal control (NC) group; *P<0.05, **P<0.01, compared with high-fat diet (HFD) group (ANOVA). MO-A: methylophiopogonanone A.

The effects of MO-A on SREBP-1c, ACC, LDLR, and PPARα mRNA and protein expression were measured by qRT-PCR and western blot analysis. As shown in Figures 7 and 8, the mRNA and protein expression levels of ACC and SREBP-1C in the HFD group were higher, whereas the expression levels of LDLR and PPARα in the HFD group were lower than those in the NC group (P<0.01). However, eight weeks of MO-A treatment up-regulated the expression levels of both LDLR and PPARα, and down-regulated the expression levels of both ACC and SREBP-1C (P<0.05 or P<0.01), indicating that MO-A ameliorated hyperlipidemia partly by modulating the expression levels involved in lipogenesis and lipid oxidation.

### Effect of MO-A on the activities of CPT-1 and FAO in the liver involved in lipid metabolism

**Figure 9.**
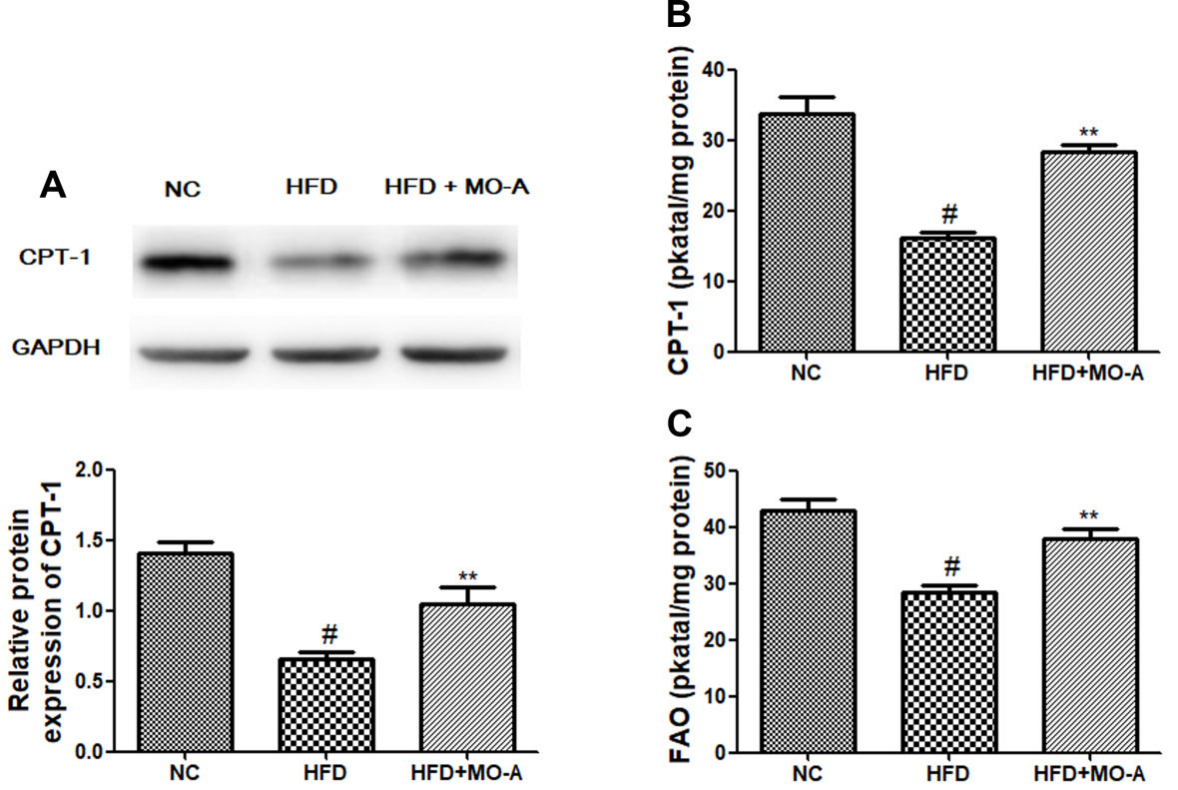
Effect of methylophiopogonanone A (MO-A) on **A**, protein expression of carnitine palmitoyltransferase-1 (CPT-1), **B**, activity of CPT-1, and **C**, activity of fatty acid oxidase (FAO) in hyperlipidemia rats. Data are reported as means±SD, n=8 rats in each group. ^#^P<0.05, compared with NC group; **P<0.01, compared with high-fat diet (HFD) group (ANOVA).

As shown in Figure 9, the protein expression level of CPT-1 in the HFD group was lower than that in the NC group (P<0.01). However, eight weeks of MO-A treatment up-regulated the expression level of CPT-1. In addition, both the activities of CPT-1 and FAO in the HFD group were decreased compared to the NC group (P<0.01). However, eight weeks of MO-A treatment increased CPT-1 and FAO activities (P<0.01), indicating that MO-A inhibited the decreased activities of CPT-1 and FAO observed in the HFD group.

## Discussion

Metabolic diseases, such as diabetes and obesity, are complications related to dyslipidemia. Drugs that alleviate hyperlipidemia are urgently needed in the prevention and treatment of cardiovascular diseases (15). Statins play an important role in stabilization of lipid-rich plaques by activating the PPARs system and are widely used to treat dyslipidemia disease (16). However, the wide application of statins is limited due to the side effects. Therefore, safe and effective pharmacological intervention for the treatment of hyperlipidemia is needed (17). Natural compounds derived from Chinese herbal medicines have been increasingly investigated to develop lipid-lowering drugs due to their lower side effects (18). MO-A is a homoisoflavonoid derived from *Ophiopogon japonicas*. According to a previous study, homoisoflavonoids isolated from *Portulaca oleracea* possess anti-adipogenic effect by the down-regulation of adipogenic transcription genes expression (19). Therefore, we speculated MO-A has a lipid-lowering effect.

A dyslipidemia model was established by feeding the SD rats with diets rich in saturated fats and cholesterol to assess the hypolipidemic effect of MO-A. Our findings indicated that the HFD group exhibited an increase in serum levels of TC, TG, and LDL-C, which was consistent with a previous report (20). In the present study, we investigated the hypolipidemic effects of MO-A for the first time. As we expected, MO-A exerted strong lipid-lowering effects in serum and hepatic lipid levels. Moreover, our results showed that MO-A reduced body, adipose tissue, and liver tissue weights. Thus, MO-A possessed the potential to prevent the initiation of pathology associated with dyslipidemia.

Hepatic lipid accumulation is deemed to be related to hyperlipidemia, one of the risk factors of cardiovascular disease (21). The liver tissue could regulate homeostasis of blood lipids by generating lipoprotein. However, excess energy of the liver could increase the incidence of insulin resistance, hyperlipidemia, and hepatic steatosis (22). Previous research revealed that regulation of the activity of hepatic lipoprotein metabolism enzymes is another therapeutic scheme for dyslipidemia (23). HL is synthesized and generated in hepatocytes, which influences the lipid composition of all lipoprotein classes by catalyzing the hydrolysis of triglycerides and phospholipids (24). Both HL and LPL play a vital role in lipoprotein metabolism. In the present study, MO-A supplementation to dyslipidemia rats improved the activities of HL and LPL. The results suggested that MO-A accelerated lipoprotein metabolism by improving the activities of HL and LPL in hyperlipidemic rats.

It is believed that HFD could induce liver damage, subsequently decreasing liver function and weakening lipid metabolism capacity (25). In the present study, we provided evidence that MO-A protected the liver against damage by increasing the serum and hepatic ALT and AST levels in the dyslipidemia rats.

The important finding of this research was that MO-A possessed a hypolipidemic effect. In the present study, we measured the genes and protein expression responsible for lipids synthesis and fatty acid oxidation (CPT-1, ACC, SREBP-1C, LDLR, and PPARα) to clarify the lipid-lowering mechanism of MO-A. SREBP-1C is a crucial transcription factor, which binds to the endoplasmic reticulum to regulate triglyceride and fatty acid synthesis (26). Moreover, SREBP-1C is involved in *de novo* fatty acid synthesis via regulating the expression of ACC (27). Previous research indicated that decreasing the expression of SREBP-1C results in a decline of the incidence of dyslipidemia and simultaneously suppresses ACC expression in C57BL/6J mice (28). Consistent with these results, our findings indicated that MO-A decreased the expression of genes and proteins related to lipogenesis of ACC and SREBP-1C. Therefore, we concluded that MO-A could alleviate hepatic *de novo* lipogenesis by decreasing the expression of ACC and SREBP-1C. In addition, the homeostasis of hepatic triglycerides is also regulated by mitochondrial oxidation. Based on this pathway, PPARα is a fatty-acid-activated nuclear hormone receptor that is involved in lipid metabolism and fatty acid oxidation (29). LDLR also plays a vital role in lipid metabolism; mRNA expression and activity of LDLR are decreased in the hypercholesterolemic rat, which showed that saturated fatty acids and high cholesterol inhibited the activity of LDLR (30). CPT-1 is an important rate-limiting enzyme involved in the mitochondrial fatty acid oxidation pathway, and FAO is a key hepatic enzyme involved in lipid metabolism (31). In present study, we observed that the expression levels of hepatic LDLR and PPARα and the activities of CPT-1 and FAO were decreased after HFD feeding, while MO-A supplementation upregulated the expression levels of CPT-1, LDLR, and PPARα and increased the activities of CPT-1 and FAO in the liver of hyperlipidemia rats. Thus, our findings implied that MO-A may also regulate disorders of lipid metabolism by CPT-1, LDLR, and PPARα, thereby decreasing the serum levels of TG and LDL-C in hyperlipidemia rats.

In conclusion, our results revealed, for the first time, that MO-A supplementation was effective in decreasing body weight and lowering lipid levels. In addition, it could prevent liver injury effectively. The protective effects of MO-A were probably associated with decreased lipid peroxidation, and increased lipolysis produced by improving LPL and HL activities. Our findings suggested that MO-A may be beneficial for the prevention and treatment of HFD-induced hyperlipidemia.
